# Cellulose particles capture aldehyde VOC pollutants†

**DOI:** 10.1039/d0ra00414f

**Published:** 2020-02-24

**Authors:** Isaac Bravo, Freddy Figueroa, Maria I. Swasy, Mohamed F. Attia, Mohamed Ateia, Domenica Encalada, Karla Vizuete, Salome Galeas, Victor H. Guerrero, Alexis Debut, Daniel C. Whitehead, Frank Alexis

**Affiliations:** School of Biological Sciences and Engineering, Yachay Tech University Urcuquí Ecuador falexis@yachaytech.edu.ec; Center of Nanosciences and Nanotechnology, Universidad de Las Fuerzas Armadas ESPE Sangolquí Ecuador; Department of Chemistry, Clemson University Clemson South Carolina USA dwhiteh@clemson.edu; Department of Bioengineering, Clemson University Clemson South Carolina USA; Department of Environmental Engineering and Earth Sciences, Clemson University Clemson South Carolina USA; Mechanical Engineering Faculty, Escuela Polytecnica Nacional Quito Ecuador

## Abstract

Aldehydes are commonly encountered Volatile Organic Compounds (VOCs) released to the atmosphere from a variety of anthropogenic sources. Based on the increasing interest in developing sustainable and environmentally friendly materials for the decontamination of VOCs, cellulose particles have emerged as one possible candidate, but there is a lack of understanding of the physicochemical properties affecting the adsorption of VOCs, and the effect of the extraction source on these intrinsic features. The present study was focused on the evaluation of unmodified cellulose particles extracted from biodiverse sources in Ecuador as potential VOC decontaminants. Modifications of the natural fibers with polyethylenimine (PEI) coating were performed to enhance the adsorption effectiveness. Fourier-transform infrared spectroscopy (FTIR), X-ray diffraction (XRD), thermogravimetric analysis (TGA), Brunauer–Emmett–Teller (BET) measurements, and scanning electron microscopy (SEM) methods were used to characterize the physicochemical properties of the isolates. Gas chromatography assays demonstrated that unmodified cellulose can adsorb an aldehyde VOC, hexanal, reaching up to a 56.42 ± 7.30% reduction. Electrostatic coating of the cellulose particles with small quantities of PEI enhanced the VOC remediation capacities (*i.e.* 98.12 ± 1.18%). Results demonstrated that the biodiverse plant source of the cellulose isolate can affect the gas capturing properties, and that these particles can be an environmentally friendly solution for effective adsorption of VOC pollutants.

## Abstract 2

Cellulose particles isolated from biodiverse sources are capable of capturing aldehyde VOCs.

## Graphical abstract

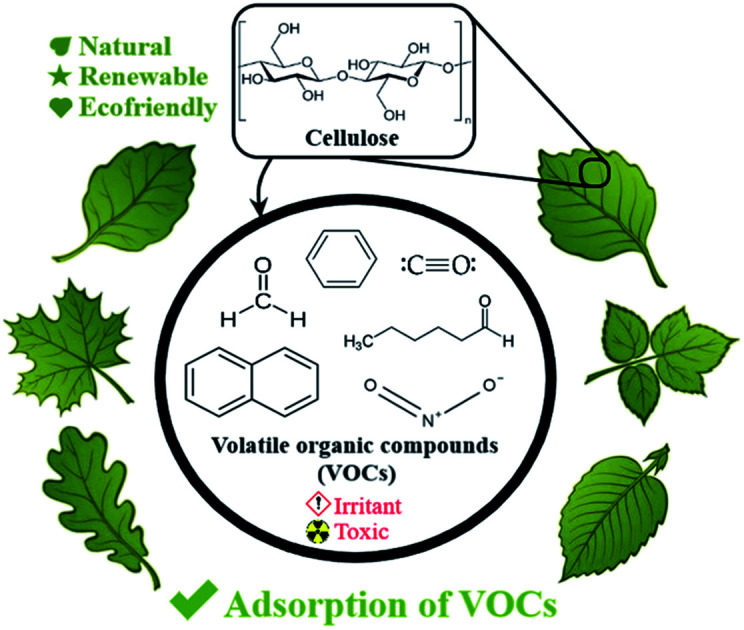

## 1. Introduction

Volatile organic compounds (VOCs) are compounds released into the atmosphere by either biogenic or anthropogenic sources such as chemical industries, agricultural operations, landfills, fuel refineries, and pharmaceutical plants.^1^ They are considered as one of the main sources of photochemical reaction in the atmosphere resulting in toxic compounds affecting the environment and human health, even when their concentration levels are within governmental regulations.^2^ To address these environmental concerns, numerous organic and inorganic materials have been developed for VOC remediation leveraging a variety of different mechanisms.^3–5^

Cellulose is the most abundant natural polymer on Earth, and can be obtained from a wide range of sources including wood, vegetation, algae, and bacteria. It is a biopolymer of β-(1,4)-linked d-glucose.^6^ Due to the presence of a large number of hydroxyl groups on the surface of the material, native cellulose can be readily functionalized by a diverse array of chemical transformations that can enhance its physicochemical properties. Cellulose particles have been of particular interest for the development of environmental remediation technologies due to their low cost, wide availability, flexibility, ease of processing, nontoxicity, and biodegradable properties.^7^ Industries and governments are beginning to pay special attention to the development of modified cellulose particles in order to use them as biological and environmental sustainable decontaminating agents.^8,9^ Thus, several studies have focused on cellulose-based materials' versatility, renewability, and functionality for many remediation applications, such as the removal of heavy metals ions in water and the adsorption of organic compounds present in polluted air.^10–12^ Only unmodified cellulose particles from wood and bacteria have been mostly used for the environmental remediation. Kim *et al.* developed activated carbon-impregnated cellulose filters as a material for removing VOCs (*e.g.* benzene, toluene, ethylbenzene) from indoor pollution. Ion *et al.* have demonstrated that bacterial cellulose might be a promising adsorbent for the removal of polar and non-polar VOCs, and proposed that blending magnetite can enhance adsorption efficiency of this biopolymer. Further, our group has studied the covalent functionalization of commercially available micro- and nano-scale cellulose with poly(ethylenimine) (PEI), resulting in materials that demonstrate the ability to capture aldehydes and carboxylic acid VOCs.^13^ Materials presenting higher surface-to-volume ratio exhibit better VOC adsorption performances due to an increase in amine sites present on the surface per unit volume.^13,14^ The chemically modified cellulose particles captured VOCs through a chemical reaction, but any intrinsic adsorption by means of non-covalent, electrostatic interactions has not been explored. More importantly, these studies are limited to commercial sources of micro- or nano-scale cellulose with or without further chemical functionalization. They did not evaluate the potential effects of the biodiverse source of the cellulose isolates on the resulting physicochemical properties of the material.

In the present study, we hypothesized that the biodiverse source of cellulose particles extracted from native plants in Ecuador might affect their physicochemical properties and in turn, the ability of the material to capture aldehyde VOCs in the gas phase. Ecuador is one of the most species-rich countries on Earth, harboring approximately twenty thousand unique species of plants from which cellulose can be extracted. We surmised that this rich biodiversity might yield cellulose particles presenting vastly different morphology, size, porosity, degradation temperatures, surface chemistry, and crystallinity. Furthermore, Ecuador possesses seventeen different ecosystems containing natural sources still undisturbed by humans as well as many endemic species of plants with undiscovered properties.^15^ This great biodiversity allowed us to evaluate a variety of plant sources that leads to cellulose particles having distinct physicochemical properties that may, in turn, improve the VOC adsorption efficiency. The ultimate goal of this study was to arrive at a better choice than commercially available cellulose previously used (*i.e.* CNC, CMC) for the development of affordable, environmentally friendly adsorbents for VOC removal. Thus, we successfully isolated novel, unmodified natural cellulose particles capable of adsorbing aldehydes VOCs. Further, we treated these natural particles with small quantities of PEI by electrostatic coating on the surface of the cellulose, thus further improving their adsorption capacity.

## 2. Experimental section

### Cellulose extraction

Cellulose extraction was carried out using established protocols^6,12,13^ of chemical extraction followed by acid/base treatment, bleaching, and multiple washings with water to remove residual chemicals. The same process was used for each sample to prevent any effect of the protocol on the cellulose properties. Control samples of cellulose microcrystals (CMC) and cellulose nanocrystals (CNC) were supplied by Sigma-Aldrich.

### PEI-coating of native cellulose

Poly(ethyleneimine) solution (PEI) (1200–1300 *M*_w_, 50 wt% in H_2_O), was supplied by Sigma-Aldrich. A 30 mg sample of unmodified cellulose isolate F28 was suspended in a 0.01% v/v PEI solution (*i.e.* 1 μL of PEI, 10 mL of H_2_O). The suspension was then placed in an ultrasonicator bath at 80 °C for 25 min. The coated cellulose materials were then collected by centrifugation and the sample was dried at room temperature for 2 d in a vacuum oven. The PEI-modified cellulose fibers were thoroughly washed with water by means of vacuum filtration.

### Physicochemical characterization

The surface structure and morphology of the different cellulose fibers were examined by using a MIRA 3 (TESCAN, CZ) field emission scanning electron microscope (FEG-SEM). X-ray diffraction (XRD) patterns were collected on an EMPYREAN diffractometer (PANalytical, NL) in a Bragg–Brentano configuration at 40 kV and 45 A and monochromatic X rays of Cu K-α wavelength (*λ* = 1.541 Å) using a Ni filter. The crystallinity index (CrI) was identified for each cellulose samples following the method described by Segal *et al.* using the eqn (1):1
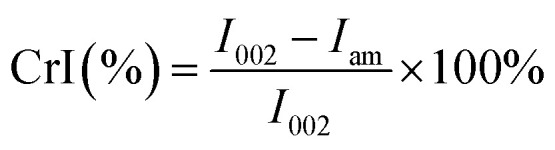
where, *I*_002_ is the maximum intensity of the 002 lattice diffraction peak and *I*_am_ is the intensity shown by the amorphous part of the cellulose samples. Lyophilized cellulose samples were mounted on a zero-background substrate. Scans were obtained from 5 to 90 degrees in 0.01 degree steps, 10 seconds per step, 16 spinner revolution time by minute, repeated eight times. CrI was calculated from the height ratio of the (200) peak and the height of the minimum between the 200 and 110 peaks.^15^ FT-IR data was recorded using a Spectrum Spotlight 200 FT-IR instrument (PerkinElmer, USA). First a spectra of the gold-plated sample holder was acquired as background, and then the spectra of the samples were recorded. The wavelength range for the analysis was between 4000 to 500 cm^−1^ with a total number of scans of 36 and a 4 cm^−1^ wavelength resolution. Thermogravimetric analysis (TGA) was used to determine the thermostability of the unmodified and PEI-treated cellulose using a Q500 instrument (TA Instruments, USA) under nitrogen over the temperature range of 27 °C to 600 °C with a heating rate of 10 °C min^−1^. Nitrogen gas adsorption was performed at 77 K with an ASAP 2020 analyser (Micromeritics Instrument Corp. U.S.) and pore size distributions were determined using density functional theory (DFT) and the calculated surface areas were from the Brunauer–Emmett–Teller (BET) equation.

### Gas chromatography assays

GC analyses replicates were carried out on cellulose samples three times each, as described in our previous studies.^1,2,13,19^ Hexanal was purchased from Sigma-Aldrich and used without purification. Agilent Technologies Gas Chromatography vials with septum screw-caps were used along with a Shimadzu GC-2014 gas chromatograph equipped with a Shimadzu AOC-20i autoinjector, a flame ionization detector (FID) and a 30 m × 0.25 mm × 0.25 μm Zebron ZB-WAX Plus capillary GC column.

## 3. Results

Based on the low cost, availability, and renewability of cellulose and Ecuadorian megabiodiversity, we chemically extracted unique unmodified cellulose particles from six different natural sources using the same protocol for each source of fibers to prevent the effect of processing on the particle extraction.^6,12,13^ Six different unmodified cellulose particles were successfully obtained from Tuna fruit from the family *Opuntia* (F17), *Passiflora tripartita*, commonly known as Taxo fruit (F19), *Chuquiragua jussieui* plant (F20), Higo fruit obtained from *Ficus carica* (F25), *Psidium guajava*, regionally known as Guava fruit (F27), and *Borojoa patinoi* or Borojo fruit (F28). Most of the sources of cellulose particles were selected due to their native properties from the Americas and Andes region (*i.e.*, *Ficus caraca* is native to Asia). The physicochemical properties of the cellulose particles were compared with commercially available cellulose microcrystals (CMCs) and cellulose nanocrystals (CNCs) purified from cotton linters, in order to highlight the unique natural properties present in native cellulose and to facilitate VOC adsorption capacity comparisons.

### Characterization of commercially available cellulose particle controls (CMC and CNC)

**Fig. 1:**
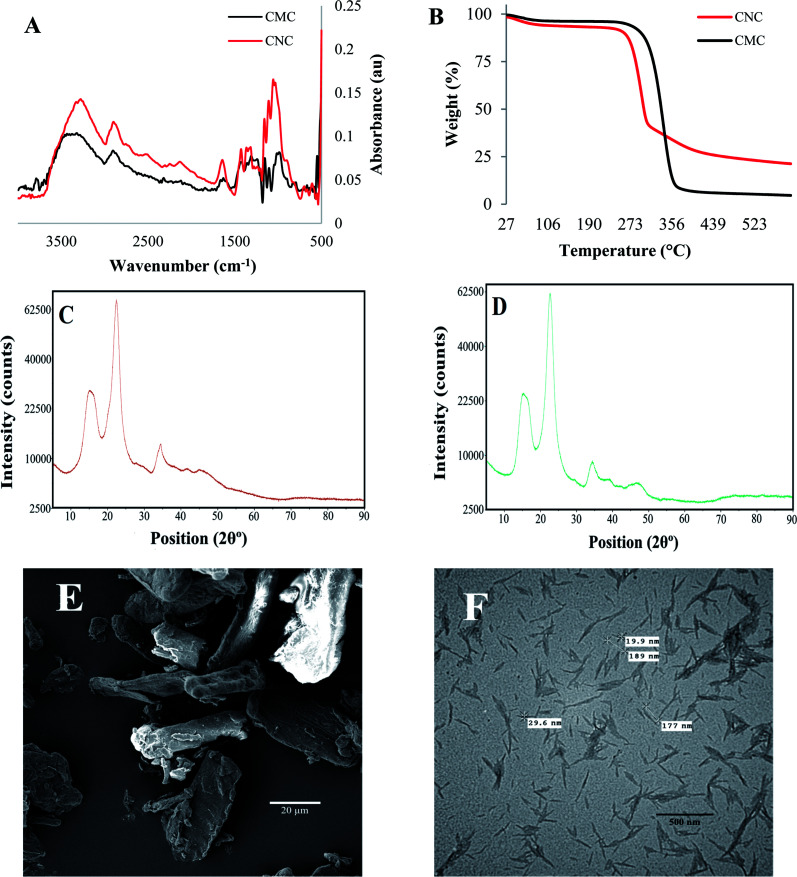
(A) FTIR spectra of the cellulose crystals (CMC and CNC controls), (B) thermal stability comparison of the controls. XRD graphs of (C) CMC and, (D) CNC. (E) SEM micrograph of CMC, and (F) TEM image of CNC.

The Fourier transform-infrared (FTIR) spectra of commercial samples of cellulose microcrystals (CMC) and cellulose nanocrystals (CNC), illustrated in Fig. 1A, indicate characteristic peaks for cellulose: C–C, C–OH, C–H ring and side group vibration bands ∼1000 cm^−1^, C–O–C glycosidic ether bands at ∼1105 cm^−1^. Additionally, important peaks are evident at ∼1300 cm^−1^, ∼1600 cm^−1^, ∼2900 cm^−1^, and ∼3300 cm^−1^ which correspond to CH_2_ rocking vibrations at C6, OH bending, sp^3^ C–H stretching, and OH stretching frequencies, respectively. Slight differences were apparent in the two samples, however. In the CNC spectrum, the C–C ring breathing, OH bending and CH_2_ rocking vibrations at C6 are marginally displaced to the left at ∼1050 cm^−1^, ∼1314 cm^−1^ and ∼1630 cm^−1^, respectively, while the peak corresponding to C–O–C group is at the same wavenumber when compared with the CMC spectrum. This confirms that the commercial, control cellulose particles are mainly composed of cellulose and do not contain hemicellulose or lignin residual contaminants. Thermogravimetric analyses (TGA) of commercial, controls revealed that CNC maintains more weight percentage at temperatures higher than 300 °C when compared with CMC, as shown in Fig. 1B. The crystallinity of the controls were also analyzed by X-ray powder diffraction (XRD), and the peaks observed for CMCs and CNCs graphs displayed in Fig. 1C and D are quite similar and correspond to a degree of crystallinity of 57.8% and 60.5%, respectively. Scanning electron microscopy (SEM) and transmission electron microscopy (TEM) observations showed that the CMCs presents a tube-like microstructure while the CNCs possess a flake-like nanostructure due to partial aggregation when preparing the sample (Fig. 1E and F).

### Characterization of cellulose from natural sources

**Fig. 2:**
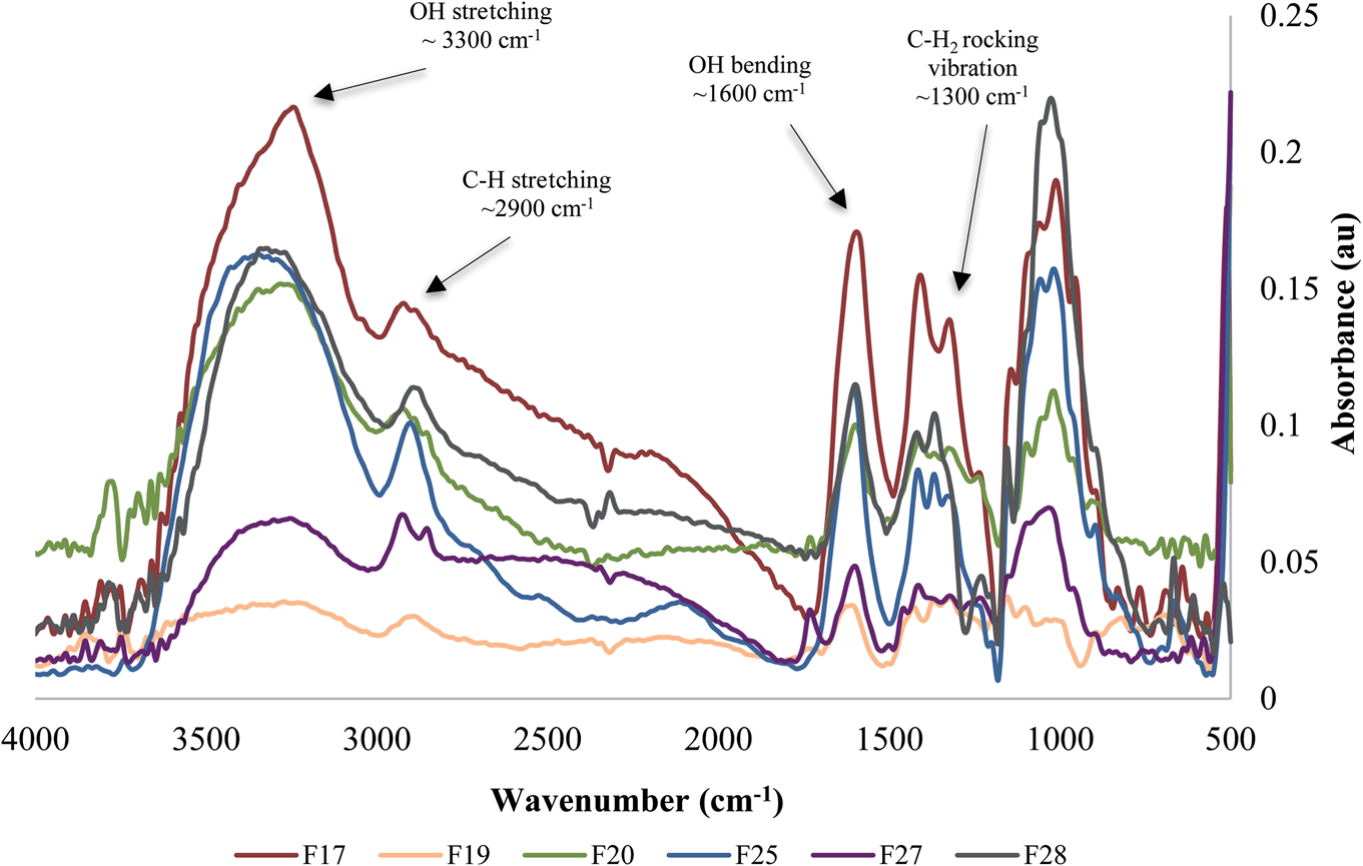
Fourier-transform infrared spectra comparison of the unmodified natural cellulose particles obtained from biodiversity.

All of the natural cellulose particles were extracted from different plant sources using the same extraction process. Each cellulose isolate exhibited unique physicochemical properties, as shown in Fig. 2. FTIR spectra were useful to identify cellulose content and residual molecules of cellular wall components, such as hemicellulose or lignin. In addition, Fig. 2 indicates that each fiber isolate (*i.e.* F17, F19, F20, F25, F27, F28) presents similar FTIR spectra with characteristics peaks similar to the commercial controls, CNC and CMC (*cf.*Fig. 1).

**Fig. 3:**
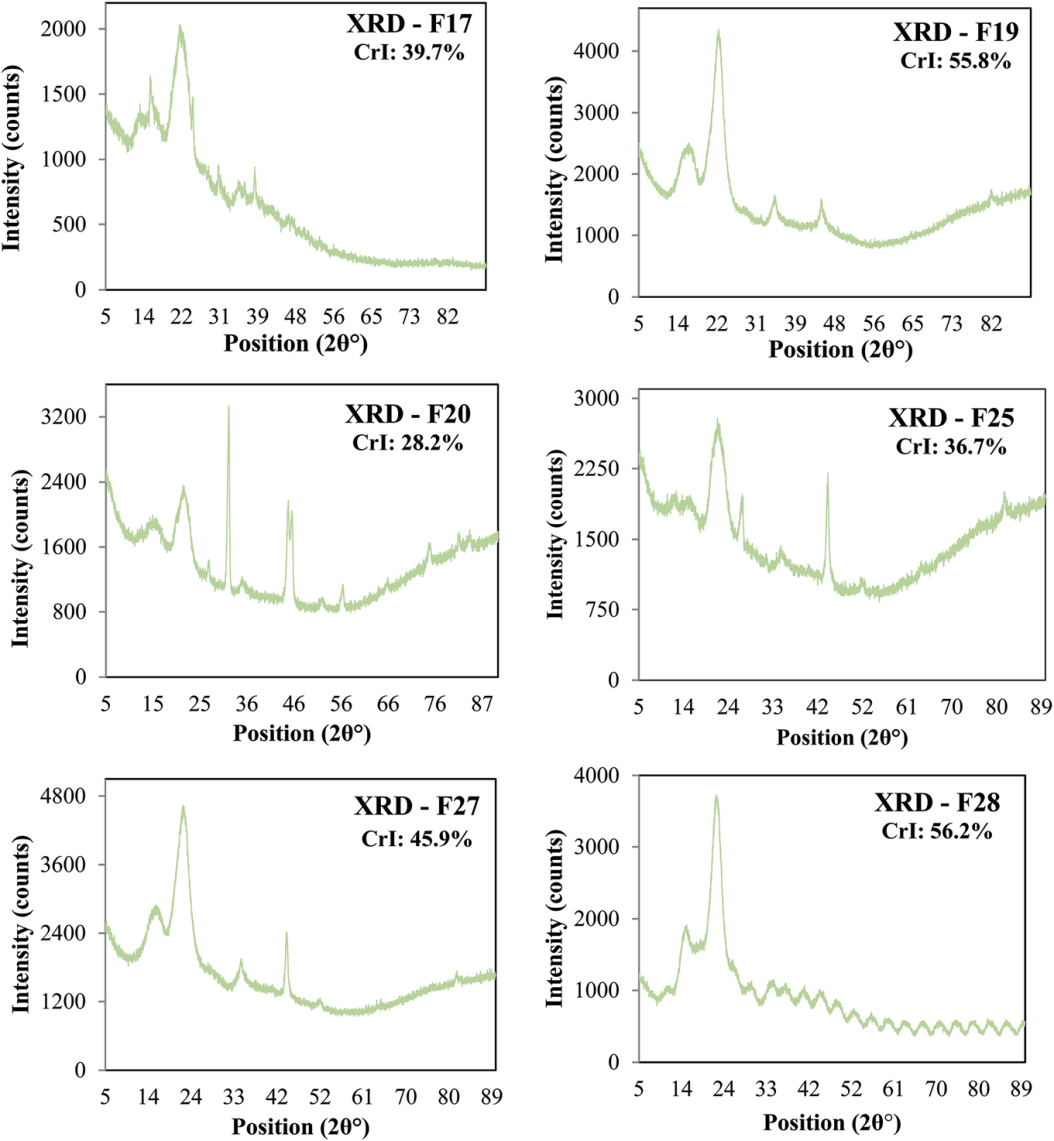
X-ray diffraction analysis and Crystallinity Index (CrI) of the natural cellulose particles: F17, F19, F20, F25, F27 and F28.

Powder X-ray diffraction (XRD) analyses of the six cellulose isolates depicted distinct graphs and different degrees of crystallinity for each, as shown in Fig. 3. The major peaks around 2*θ* = 20–25° are attributed to cellulose crystalline structure, while the peaks around 2*θ* = 15–18°, represents the samples' amorphous region.^16^

**Fig. 4:**
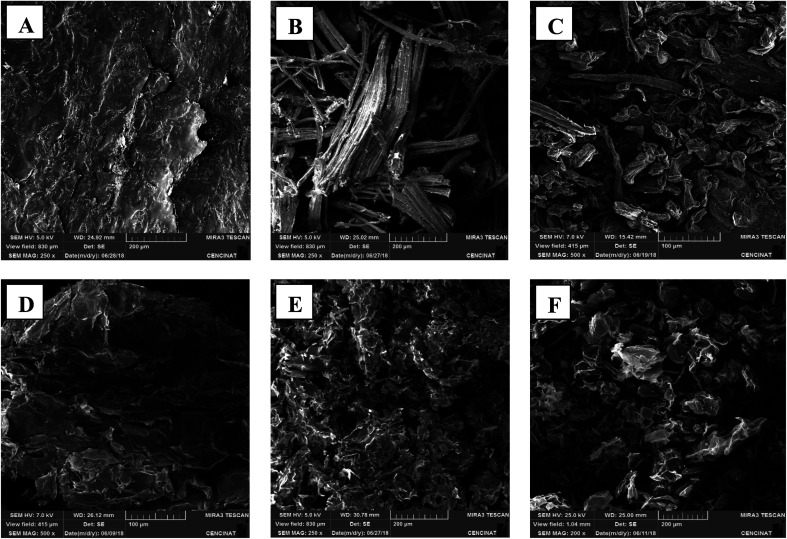
SEM micrographs of the natural unmodified cellulose particles: (A) F17, (B) F19, (C) F20, (D) F25, (E) F27, and (F) F28.

Scanning electron microscopy (SEM) observations on the new isolates reveal that each cellulose sample presents a unique morphology, porosity, and size despite the fact that the same extraction process was used for each sample, suggesting that the unique sample morphology is related to its plant source (Fig. 4).

**Fig. 5:**
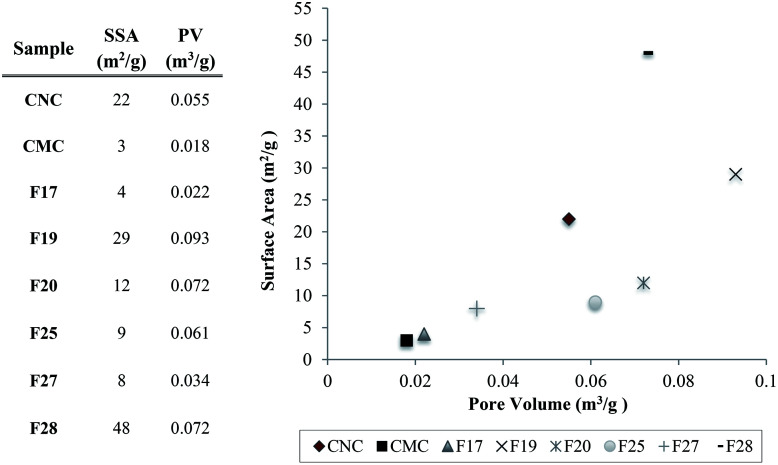
Pore volume (PV) and sample surface area (SSA) table of the cellulose samples, and the distribution of the pore volume correlated with the surface area of the particles and the controls.

The BET characterization results indicate that while the particles are closely related, small differences are still apparent owing to the intrinsically different physicochemical properties arising from the unique natural sources of the samples. Additionally, samples surface areas (SSA) and pore volume (PV) are illustrated in Fig. 5, as well as the correlation between surface area and pore volume of samples. We found that the BET analysis of our extracted cellulose materials (Fig. 5) is in agreement with different types of cellulose reported previously.^17^ In addition, for example, F28 (SA = 48) showed higher capture efficiency of hexanal vapors over both the commercial CMC (SA = 3) and CNC (SA = 22), highlighting the significance of the direct correlation between the surface area and adsorption capacity and kinetics.

### Characterization of PEI-modified cellulose particles F28

**Fig. 6:**
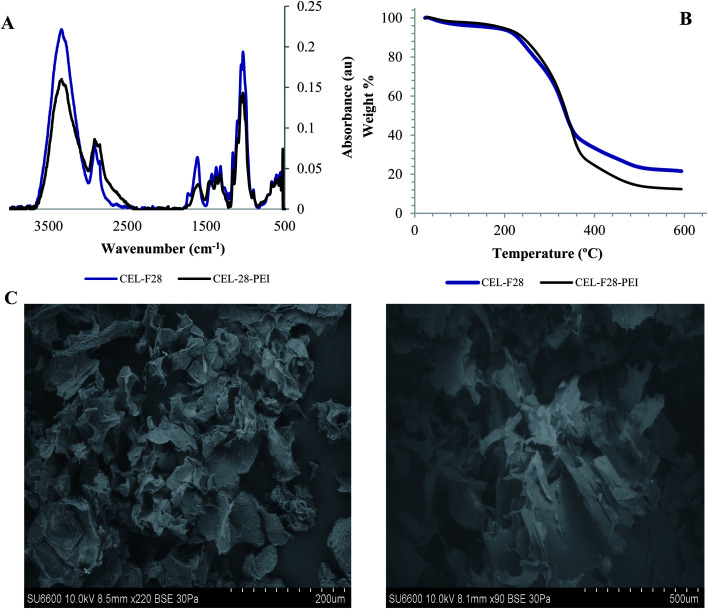
(A) FT-IR spectra of unmodified and PEI-treated cellulose particle F28, (B) TGA analyses of isolated F28 before and after PEI surface coating, and (C) SEM micrographs of natural and PEI-treated F28 samples, respectively.

In order to surface-modify cellulose particle F28 with amine functional groups to facilitate the covalent absorption of aldehyde VOCs,^13^ the native isolate was coated with PEI upon ultrasonication with a 0.01% v/v PEI solution. The presence of PEI was easily confirmed by the emergence of new N–H bending resonances around ∼1550 cm^−1^ in the infrared spectrum (Fig. 6A). Thermogravimetric analyses of F28 and F28-PEI are illustrated in Fig. 6B, and provide additional evidence for the successful electrostatic coating of the fiber using small amounts of PEI. The thermal degradation of the unmodified F28, initiates at 229 °C. PEI coating on the surface of cellulose particle F28 causes the thermal degradation of the material to initiate at slightly warmer temperatures (240 °C), (Fig. 6B). Moreover, the weight loss curves for F28 and F28-PEI present important differences in the final values of the graph, which indicates the efficiency of the electrostatic coating of F28 with PEI. The thermodegradation profiles of both F28 and PEI-coated F28 are consistent with cellulose materials reported elsewhere supporting our results.^18^ Structural and morphological nuances are observable through SEM images (Fig. 6C). It is notable that the unmodified F28 isolate and its PEI-modified congener present similar, “shriveled” structures. Nevertheless, there are a few differences in their overall morphologies; F28-PEI displays flat and folded, rugged surfaces that manifest in tube-like structures due to the presence of the amine coating. In contrast, the unmodified F28 isolated shows many uneven and pleated surfaces.

### Gas chromatography assays

**Fig. 7:**
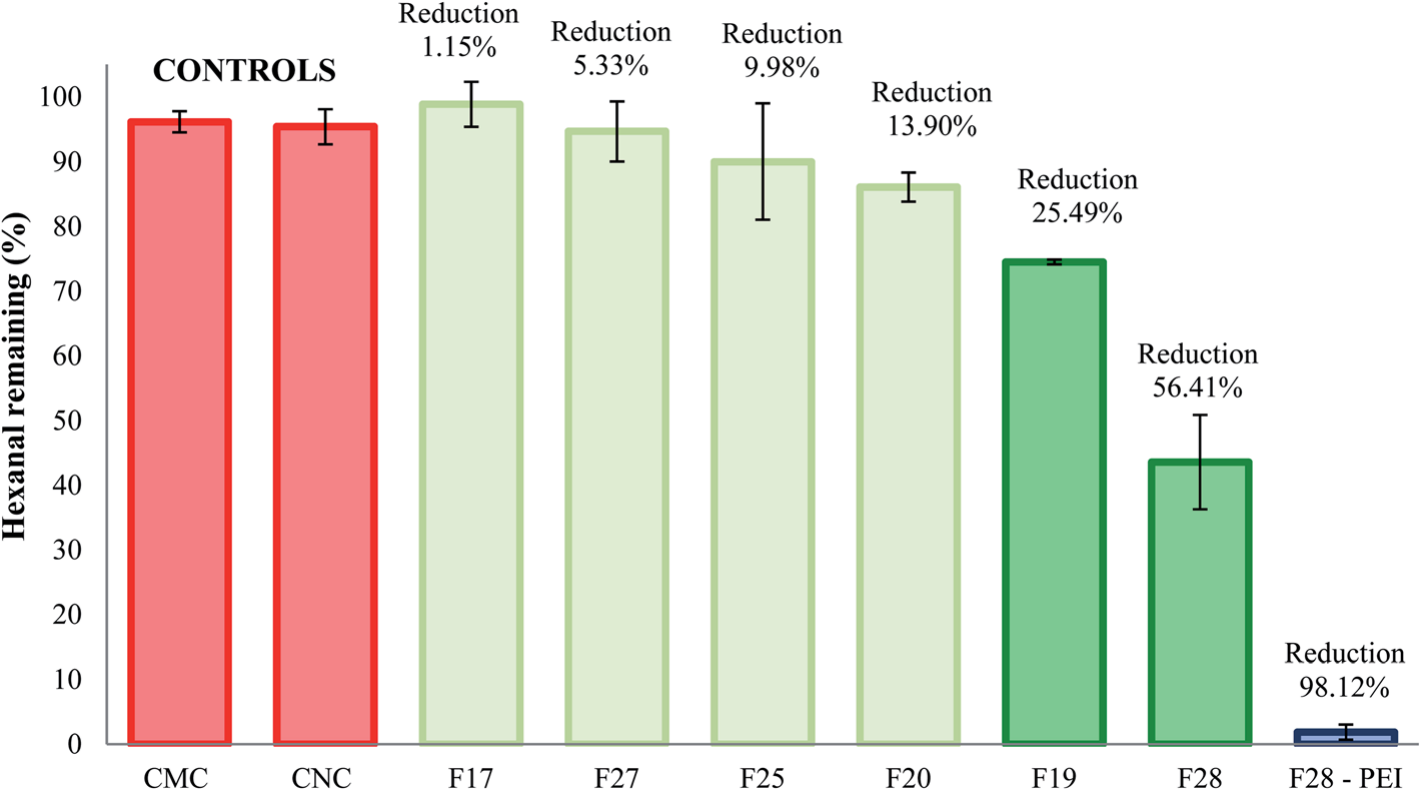
Percent reduction of hexanal vapors after treatment with cellulose crystals controls and unmodified or PEI-treated natural cellulose samples.

After the successful isolation and characterization of a series of cellulose particles, we conducted gas chromatography experiments to assess the VOC absorption efficiency of the particles and a PEI-coated congener using a model aldehyde VOC, hexanal. Gas chromatography assay results after 30 min exposure of the particles to hexanal vapors are shown in Fig. 7. The VOC absorption efficiency of the commercially available CMC and CNC samples are also included as controls. The results demonstrated that unmodified F19 and F28 cellulose exhibited significantly higher capturing capacities than the commercially available cellulose controls. These cellulose samples were capable of effecting a 25.49 ± 0.36% and 56.42 ± 7.30% reduction of hexanal vapors, respectively. For comparison, the commercial CMC and CNC samples performed a VOC reduction of 3.87 ± 1.64% and 4.60 ± 2.68% respectively. The latter results are similar (*i.e.* no statistical differences) to the performance of isolates F17, F20, F25 and F27, which showed absorption capacities of 1.15 ± 3.47%, 13.90 ± 2.26%, 9.98 ± 8.97% and 5.33 ± 4.62%, respectively.

We have previously demonstrated that surface modification of particles to display reactive amine functional groups can increase the VOC remediation properties for cellulosic, polymeric, and clay-based materials.^19^ Indeed, PEI-coated F28 sample results (also depicted in Fig. 7) demonstrated excellent adsorption performance in the presence of hexanal vapors, leading to a 98.12 ± 1.17% reduction of the target VOC.

## 4. Discussion

Cellulose is a natural polymer available worldwide from a diverse array of biological sources, which the literature has determined as an environmental sustainable material. Six unmodified cellulose particles were successfully extracted from natural plant sources native to Ecuador, and the particles appear to have unique physicochemical properties compared to commercial cellulose samples. These particles were extracted using the exact same process and characterized by a combination of FT-IR spectroscopy, X-ray diffraction, BET measurements, and SEM. Importantly, the FT-IR analyses indicate that the chemical composition of the particles are quite similar to commercial cellulose controls. These data indicate that the particles are chiefly comprised of pure cellulose with little contamination from other biopolymers including hemi-cellulose and lignin. According to the XRD results, the diffraction patterns show a typical cellulose configuration with a crystalline (2*θ* = 20–25°) and amorphous part (2*θ* = 15–18°). Therefore, the variation of the crystallinity index between the samples is likely due to the plant source of the cellulose, since all samples were extracted using the same protocol. SEM images of cellulose particles from different natural sources exhibited very unique morphologies and structures. This fact confirms that the degree of porosity, shape, and size differ for every cellulose sample extracted from different natural sources. Further, characterization of F28-PEI revealed that the cellulose particles could be successfully surface-coated with PEI to display reactive amine functional groups for the covalent capture of VOCs. Previous ^1^H NMR spectroscopy analysis carried out in past studies demonstrated the formation of imine bonds resulting from the interaction between PEI-NPs and pivaldehyde, thus successfully proving the potential capture mechanism of aldehydes using PEI-modified materials.^19^ Evidence of the poly(amine)-coating was provided by FT-IR spectroscopy and TGA. According to SEM analyses of PEI-modified and non-modified F28, the presence of the PEI polymer may alter the surface morphology of the sample.

Gas chromatography experiments revealed that some of the natural unmodified cellulose particles (*i.e.* F19 and F28) demonstrated a significant, intrinsic ability to absorb hexanal vapors (*i.e.* up to ∼57% adsorption for F28) as compared to the absorption capability of commercially available cellulose microcrystal and nanocrystals controls (*i.e.* CMC and CNC). SEM and BET analyses showed that the degree of porosity of the specific cellulose isolate plays a critical role in the VOC adsorption capacity of the material. The two samples with the greatest capacity for VOC adsorption, isolates F19 and F28, had larger pore volumes (0.093 and 0.072 m^3^ g^−1^, respectively) than the other isolates and the commercial CMC/CNC samples (≤0.061 m^3^ g^−1^). The overall surface area of the cellulose particle also plays an important role in the VOC adsorption efficiency. Samples F19 and F28 possess surface areas of 28 m^2^ g^−1^ and 48 m^2^ g^−1^ surface areas, respectively, which are greater than the surface areas observed for the less-effective F17, F20, F25 and F27 isolates. Nevertheless, the degree of porosity of the particle emerged as the main factor affecting the VOC adsorption capability of the cellulose samples. Note that the commercially available controls CMC and CNC, which presented poor VOC adsorption capacity, exhibit small pore volumes. Particularly compelling, the CNC control effected poor VOC adsorption, despite having a rather large surface area of 22 m^2^ g^−1^ while lacking sufficient pore volume (0.055 m^3^ g^−1^) for optimal VOC capture. The addition, by electrostatic coating, of small amounts of PEI polymer onto the surface of cellulose particle F28 greatly increased the aldehyde VOC removal capacity of the parent materials, obtaining a performance of a 98% reduction of hexanal vapors.

The results presented in this study clearly indicate that the physicochemical features that each cellulose sample present (*i.e.* surface area, surface morphology, and pore volume) directly affects the efficiency of VOC remediation. It was reported in the literature that the surface area-to-volume ratio and porosity parameters plays an important role in the efficiency of materials for the remediation of organic pollutants in water and air.^11,19,20^ Nonetheless, the correlation for absorption of hexanal vapors is not linear. The twin features of large surface area coupled with high pore volume of cellulose particles F19 and F28 as compared with commercially available controls makes these particles uniquely effective for the capture of VOCs. The magnitude of the surface area can determine the density and pore distribution on the surface of the materials, thus explaining why isolates with large pore sizes but superficial surface areas perform poorly in VOC capture studies.

## 5. Conclusions

In this study, six novel unmodified cellulose isolates were successfully extracted and purified from different natural plant sources of Ecuadorian biodiversity. Intensive physicochemical characterization was performed on cellulose isolates using FTIR, XRD, SEM and BET, and indicated that the natural source of extraction directly affects the physicochemical properties of the isolates. These properties, most notably the varying degree of porosity, in turn directly affected the intrinsic VOC decontamination capabilities of the isolates. More importantly, samples F19 and F28 exhibited a better VOC absorption performance due to their specific intermediate surface area and pore volume parameters, which are critical factors that directly affect the adsorption capacities of cellulose fibers. Higher surface area results in larger degrees of porosity, which in turn yields better VOC adsorption efficiency. This study demonstrated that there are optimal physicochemical properties of cellulose that directly affect the intrinsic ability of the material to capture VOCs. Additionally, the surface coating of cellulose isolates with small quantities of PEI greatly increased the absorption capacity of the samples, facilitating the capture of hexanal vapors up to 98%. Thus, electrostatic coating of cellulose fibers results in an efficient manner to modify cellulose, avoiding several chemical steps and the use of a wide variety of reagents. Ultimately, this study demonstrates the exciting potential for mining the inherent biodiversity of ecologically rich locales such as Ecuador in order to generate unique and structurally diverse cellulosic platforms for environmental applications.

## Author contributions

F. A. and D. C. W. conceived and designed most of the experiments, analysed the research, and wrote part of the manuscript text. I. B., F. F., and D. E. performed most of the experiments, analysed data, and wrote the main manuscript text. M. S. helped perform all gas chromatography experiments. M. A., M. At., S. G., V. H. G., K. V., A. D. helped perform part of the characterization experiments. All authors reviewed, discussed, and commented on the manuscript.

## Conflicts of interest

The authors declare no competing interests.

## Supplementary Material

RA-010-D0RA00414F-s001

## References

[cit1] Klimont Z., Streets D. G., Gupta S., Cofala J., Lixin F., Ichikawa Y. (2002). Atmos. Environ..

[cit2] (b) Whitehead D. C. and Alexis F., *US Pat.*, 20160220952, 2016, http://www.freepatentsonline.com/y2016/0220952.html

[cit3] (d) Anjum N. A. , in Enhancing cleanup of environmental pollutants, ed. N. A. Anjum, S. S. Gill and N. Tuteja, Springer International, 2017, vol. 1, pp. 289–315

[cit4] Khin M. M., Nair A. S., Babu V. J., Murugan R., Ramakrishna S. (2012). Energy Environ. Sci..

[cit5] Das S., Sen B., Debnath N. (2015). Environ. Sci. Pollut. Res. Int..

[cit6] Bethke K., Palantöken S., Andrei V., Roß M., Raghuwanshi V. S., Kattemann F., Greis K., Ingber T. T. K., Stückrath J. B., Valiyaveettil S., Rademann K. (2018). Adv. Funct. Mater..

[cit7] Gong X., Huang D., Liu Y., Peng Z., Zeng G., Xu P., Cheng M., Wang R., Wan J. (2018). Crit. Rev. Biotechnol..

[cit8] Thakur V. K., Thakur M. K. (2014). Carbohydr. Polym..

[cit9] Zhou S., Nyholm L., Strømme M., Wang Z. (2019). Acc. Chem. Res..

[cit10] Wan Ngah W. S., Hanafiah M. A. K. M. (2008). Bioresour. Technol..

[cit11] Wu Y., Pang H., Liu Y., Wang X., Yu S., Fu D., Chen J., Wang X. (2019). Environ. Pollut..

[cit12] Chen Q., Zheng J., Wen L., Yang C., Zhang L. (2019). Chemosphere.

[cit13] Guerra F. D., Campbell M. L., Whitehead D. C., Alexis F. (2017). ChemistrySelect.

[cit14] Kim S. Y., Yoon Y. H., Kim K. S. (2016). Int. J. Environ. Sci. Technol..

[cit15] (a) Valencia R. , Biodiversity and Conservation of Neotropical Montane Forests, Proc. Symposium, 1995, p. 239

[cit16] Segal L., Creely J. J., Martin A. E., Conrad C. M. (1959). Text. Res. J..

[cit17] Lapina V. A., Akhremkova G. S. (2006). Russ. J. Phys. Chem. A.

[cit18] Leal G. F., Ramos L. A., Barrett D. H., Curvelo A. A., Rodella C. B. (2015). Thermochim. Acta.

[cit19] Campbell M. L., Guerra F. D., Dhulekar J., Alexis F., Whitehead D. C. (2015). Chem.–Eur. J..

[cit20] Ruminski A. M., Jeon K. J., Urban J. J. (2011). J. Mater. Chem..

