# Supplementary material for: Cellulose particles capture aldehyde VOC pollutants
Source: RSC Adv. 2020 Feb 24;10(13):7967–75. doi: 10.1039/d0ra00414f (PMC9049895; doi:10.1039/d0ra00414f)
Supplement: RA-010-D0RA00414F-s001 [file RA-010-D0RA00414F-s001.pdf]

Electronic Supplementary Material (ESI) for RSC Advances.
This journal is © The Royal Society of Chemistry 2020

Cellulose Particles Capture Aldehyde VOC Pollutants

Isaac Bravo[1], Freddy Figueroa[1], Maria I. Swasy[3], Mohamed F. Attia[4], Mohamed Ateia[5], Domenica Encalada[1], Karla Vizuete[2], Salome Galeas[6], Victor H. Guerrero[6], Alexis Debut[2], Daniel C. Whitehead[3,], and Frank Alexis[1,#,*]*

Supplementary Figure

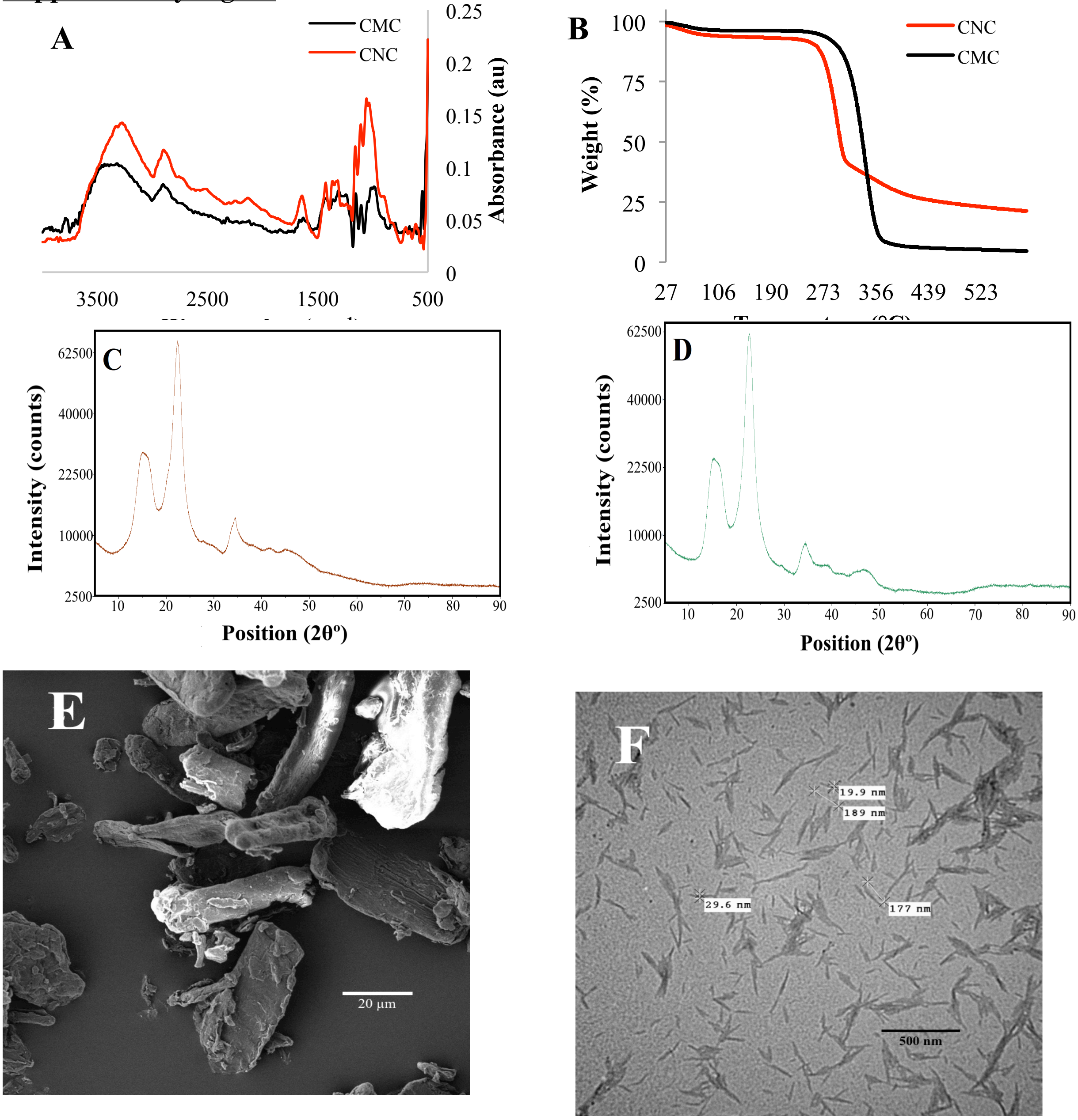

Supplementary Figure 1. A) FTIR spectrum comparison of the cellulose crystals (CMC and CNC controls), B) Thermal stability comparison of the controls. XRD graphs of C) CMC and, D) CNC. E) SEM micrograph of CMC, and F) TEM image of CNC.
